# A Retrospective Analysis of Maxillofacial Trauma in Shiraz, Iran: a 6-Year- Study of 768 Patients (2004-2010)

**Published:** 2014-03

**Authors:** HR. Arabion, R. Tabrizi, E. Aliabadi, M. Gholami, K. Zarei

**Affiliations:** a Dept. of Maxillofacial Surgery, School of Dentistry, Shiraz University of Medical Science, Shiraz, Iran; b Dept. of Maxillofacial Surgery, Hamedan University of Medical Science, Hamedan, Iran.; c Postgraduate student Dept. of Maxillofacial Surgery, Shiraz University of Medical Science, Shiraz, Iran.

**Keywords:** Trauma, Mandible, Fracture, Maxilla, Facial

## Abstract

**Statement of Problem: **Information about the etiology and incidence of maxillofacial trauma is important for prevention and appropriate treatments of such injuries.

**Purpose:** The purpose of this retrospective study was to conduct an analysis of maxillofacial injuries transferred and/or referred to the department of maxillofacial Surgery at Chamran emergency hospital, Shiraz, over a 6-year period with special reference to age, gender, occupation, date, type, site, etiology and clinical management.

**Materials and Method:** The data for this study were collected and reviewed retrospectively from the records and radiographs of 768 patients who were treated for maxillofacial trauma in the department of maxillofacial surgery at the Shiraz Chamran Emergency Hospital, Iran, between 2004 and 2010.

**Results: **A total of 730 of the subjects were the patients with fractures of the facial skeleton. The mean age was 26.6± 12.6 years, ranging from 2 to 81 years. Traffic accident was the most frequent etiological factor of maxillofacial fractures irrespective of gender (69.9% for men and 54.2% for women), whereas the second most frequent cause of injuries was falling down (9.8% for men and 21.5% for women) .The other etiologies were assaults (5.2%), sport related injuries (1.3%) and firearm injuries (1%). Regarding the head injuries in patients with maxillofacial fractures, brain contusion was seen in 227(29.6%) patients and 13.5% of patients had lacerations in the facial soft tissue. The monthly distribution peaked in October, with 81 cases (10.5%), which would be for the reason that schools open in this month. The next highest incidence was in December, with 80 cases (10.4%), probably because of the changing weather's effect on road traffic.

**Conclusion: **Isolated mandibular fracture due to the road traffic accident was the most common type of maxillofacial injuries in the city of Shiraz.

## Introduction

Maxillofacial injuries frequently occur in acutely traumatized patients. Changes to the facial skeleton distort the patient's appearance and may compromise the function of several structures including the masticatory system, ocular system, olfactory apparatus and nasal airway. There are many causes of facial fractures and there is much variability depending on cultural, economic, social and religious variance of the examined population [1]. This variable influences the distribution of the etiological factors that are seen in maxillofacial units around the world [2]. Periodic verification of the etiology of maxillofacial injuries helps to assess the proficiency of road safety measures such as speed limits, drunk driving and seat beat belt laws. It also helps in evaluating the behavioral patterns of the people in different countries

and it helps recommend other ways in which injuries to the face can be averted [3]. The purpose of this retrospective study was to analyze the maxillofacial injuries transferred and/or referred to the department of maxillofacial surgery at the Chamran emergency hospital, Shiraz, over a 6-year period, with special reference to the age, gender, occupation, date, type, site, etiology and clinical management.

## Materials and Method

The data for this study were collected and reviewed retrospectively from the records and radiographs of 768 patients who were treated for maxillofacial trauma in the department of maxillofacial surgery in Shiraz Chamran emergency hospital, Iran, between 2004 and 2010. The first year postgraduate students were responsible for data collection from the patients. The source of data was the patient radiographs and the performed clinical examination. The classification of fractures was done based on the Fonsceca definition as follows [4]:

1. Midline: fractures between central incisors
2. Para-symphyseal: fractures occurring within the area of the symphysis.
3. Symphysis: bounded by vertical lines distal to the canine teeth.
4. Body from the distal symphysis to a line coinciding with the alveolar border of the masseter muscle.
5. Angle: triangular region bounded by the anterior border of masseter to the postero-superior attachment of the masseter muscle
6. Ramus: bounded by superior aspect of the angle to two lines forming an apex at the sigmoid notch.
7. Condylar process: area of the condylar process superior to the ramus region.
8. Coronoid process: include the coronoid process of the mandible superior to the ramus region.
9. Alveolar process: the region that would normally contain teeth.

The data recorded included name, age, gender, date, occupation, consciousness, cause of injury, site, type of operation(s) and head trauma.

## Results

**Figure 1:**
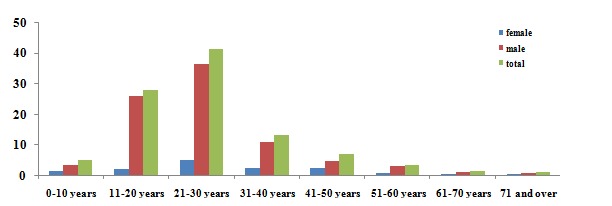
The diagram shows the age distribution of patients

During the 6 years of study, 768 patients were hospitalized and treated. There were 660 males (86%) and 107 females (14%), with a male to female ratio of 6.1:1. A total of730 of subjects were patients with fractures of the facial skeleton. The mean age was 26.6±12.6 years, ranging from 2 to 81years. The patients’ age distribution is shown in Figure 1.

**Figure 2:**
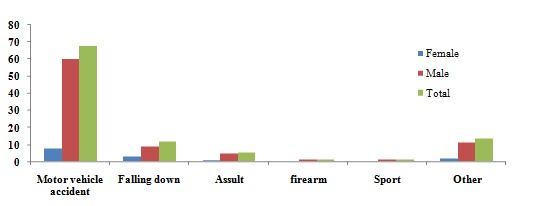
The causes of injuries in the traumatic patients

The causes of injuries are listed in Figure 2. Traffic accident was the most frequent etiological factor in maxillofacial fractures regardless of gender (69.9% for men and 54.2% for women), whereas the second most frequent cause of injuries was falling down (9.8% for men and 21.5% for women). The other etiologies maintained a similar hierarchy, including: assault (5.2%), sports related injuries (1.3%) and firearm injuries (1%).

According to the investigation of socioeconomic activity, 56.5% of the patients had professional job skill (56.2 % for men and0.3 % for women), and 17.6 % of them were students (Table 1).

**Table 1:** Socioeconomic activity of patients with maxillofacial trauma

**Occupation**	**Male (%)**	**Female (%)**	**Total (%)**
Self employment	388(50.5)	0(0)	388(50.5)
Employee	44(5.7)	2(0.3)	46(6)
Housekeeping	0(0)	80(10.4)	80(10.4)
Student	123(16)	12(1.6)	135(17.6)
Other	106(13.8)	13(1.7)	119(15.5)

In this study, 730 patients suffered from 1118 facial fractures, of which the most common was mandibular fracture with a prevalence of (448, 58.4%) followed by zygomatic complex fracture (185, 24.1%), orbital fracture (116, 15.1%) and maxillary Lefort fracture (95, 12.4%).

The most common mandibular fracture site was the body (31.5%); followed by the condyle (19.3%), the angle (16.9%), the parasymphyseal regions (16.1%), the symphysis area (10.2%), the coronoid process (4.3%) and the ramus (1.6%).The fracture sites are presented in Table 2.

**Table 2:** Site distribution of maxillofacial fractures

**Region**	**Anatomic site**	**Number of patients**	**Percent**
Upper third			
	Frontal sinus fracture	6	0.8
	Nasoethmoiedal fracture	26	3.4
Middle third			
	Nasal fracture	85	11.1
	Zygomaticomaxillary fracture	185	24.1
	Zygomatic arch fracture	4	0.5
	Orbital fracture	116	15.1
	Maxillary fracture	95	12.4
	Lefort I	46	6
	Lefort II	33	4.3
	Lefort III	16	2.1
Lower third			
	Isolated mandibular fracture		
	Condyle	49	6.4
	Coronoid	11	1.4
	Ramus	4	0.5
	Angle	43	5.6
	Body	80	10.5
	Para-symphysis	41	5.3
	Symphysis	26	3.4
	Multiple mandibular fracture	194	25.3
Other			
	Dentoalveolar fracture	57	7.4

The data regarding head injuries in patients with maxillofacial fractures demonstrated that brain contusion was seen in 227(29.6%) patients and 13.5% of patients had lacerations in the facial soft tissue. The monthly distribution peaked in October, with 81 cases (10.5%), which seems to have been due to the schools’ opening or the changing weather’s effect on road traffic (Table 3). 696 patients (90.6%) were treated as inpatients with a mean period of hospitalization of 6.5 days, opening.

**Table 3:** Monthly distribution of maxillofacial trauma patients

** **	**January**	**February**	**March**	**April**	**May**	**June**	**July**	**August**	**September**	**October**	**November**	**December**
Number	48	56	35	62	67	76	62	65	71	81	65	80
Percent	6.3	7.3	4.6	8.1	8.7	9.9	8.1	8.5	9.2	10.5	8.5	10.4

The next highest incidence was in December, with 80 cases (10.4%), probably because ranging from 1 to 41 days. Patients considered ‘‘untreated’’ accounted for 72 cases (9.4%), including refusal of treatment, death, and institution transfers. The surgical treatment details of 696 patients are shown in Table 4.

**Table 4:** Surgical treatment modalities according to the site of maxillofacial fractures (Fx) (percent)

	**Mandibular** **Fx**	**Maxillary** **Fx**	**ZMC** **Fx**	**Zygomatic** **Arch Fx**	**Nasal** **Fx**	**Frontal** **Fx**	**Orbital** **Fx**	**NOE Fx**	**Dento** **alveolar Fx**	**Total**
Open reduction	69.9	75.8	83.2	0	0	50	87.9	84.6	61.4	68.8
Close reduction	25	13.7	3.2	100	88.2	16.7	0.9	3.8	35.1	21.9
No treatment	5.4	10.5	13.5	0	11.8	33.3	11.2	11.5	3.5	9.4

Patients were under routine followed-up for up to 1 month after treatment in cases of simple reduction and fixation surgery. Additional follow-up was considered  if any complications were encountered in the patients. Of the 768 patients included in this study, 103(13.4%) showed some degree of postoperative malocclusion that was managed with elastic therapy. Ankylosis was developed in 27 patients (3.5%) and was managed with physiotherapy.

## Discussion

Trauma is the leading cause of death in the first 40 years of life. Traumatic injury could be considered as an etiology of productivity loss, causing more loss of working years than heart disease and cancer combined. Fractures of the facial skeleton are a common part of the multiple traumas resulting from the motor vehicle crashes and industrial accidents, as well as the sports and assaults [4]. In most developed countries, violence has replaced vehicle collisions as the main cause of maxillofacial trauma; while, in many developing countries, road traffic accidents (RTAs) remain the major cause [5]. 50- 70% of people who survive the traffic accidents had facial trauma [6].

The etiology of facial trauma reported in this study is similar to that reported in other studies conducted in the Middle East and Africa [1, 7-11] in which RTAs were the main cause of maxillofacial fractures. The present study is also consistent with other studies from other parts of the world [12-17]. On the other hand, assault-related maxillofacial injuries were reported to be more common in developed countries [3-2, 18-21]. In our study, assault-related maxillofacial fractures constituted only 5.2% of cases .It seems that increased use of protective measures such as seat belts, airbags, motorcycle helmets and strictly enforced speed limits in developed countries has been credited with a reduction in the incidence of maxillofacial trauma due to RTAs [22].

The subjects, eligible for inclusion in the study, had a maxillofacial fracture and were referred to the Chamran hospital emergency department. The subjects were excluded from the study if their records were not completed.

Facial fractures were distributed in a fairly normal curve by age with a peak incidence occurring between ages 20 and 30.Also, children under 12 involved in 5–10% of all facial fractures .Most facial traumas in children involved the lacerations and soft tissue injuries. The reasons for the lower incidence of facial fractures in children can be concluded as the face is smaller in relation to the rest of the head, there is a lower proportion of cortical bone to cancellous bone in the children's faces, poorly developed sinuses make the bones stronger and fat pads provide protection for the facial bones [23]. As in this study, a high male-to-female ratio among maxillofacial injury victims has been widely reported [12, 1, 24, 14-15]. This is attributed to the fact that men are more involved in outdoor activities and more frequently exposed to violent interactions. Furthermore, male vehicle drivers outnumber female drivers. This ratio seems to be lower in developed countries because of the greater socioeconomic outdoor activity of women [25-26].

In this study, the isolated mandibular fracture was the most common type of maxillofacial fracture. This is consistent with findings in some other studies [7, 1, 13, 27-30, 14, 18, 31], but different from the studies that reported higher rates of zygomatic [20], nasal [32-33] or midface [34-35] bone fractures. One reason for this difference could be that most of nasal fractures are usually referred to the Namazi hospital where the ENT department is located. Moreover, the cause of trauma in our study was found to be the road traffic accident but in other studies was assault, which often leads to nasal and midface fractures. In our study, the most common site of mandibular fracture was the body of the mandible, which is in agreement with some studies [7, 13, 24, 31] but not other researches, in which the angle [36-37], condyle [15], or para-symphysis [38] was the most common site of fracture. The second most common site of mandibular fracture was condyle, followed by angle, parasymphysis, symphysis, coronoid process and ramus.

The definition of the fractures is done based on the place of the fractures, so the absence of an acceptable universal classification of fractures can lead to different result in different studies, therefore, there is a necessity for making a universal classification of facial fractures. 

The head and brain injuries are commonly associated with facial trauma, particularly the upper face. The brain injury occurs in 15- 48% of people with maxillofacial trauma [39]. Thaller SR reported a 55% incidence of concomitant facial fracture and brain injury [40]. In the current study, brain contusion was found in 29.6% of cases.

In the past 20 years, changes in maxillofacial trauma management have been strongly influenced by innovations in materials and technology [41-42], since some issues such as early recovery, segment stability and patient comfort have been considered paramount in the treatment of maxillofacial fractures [43]. The treatment of facial fractures varies from surgeon to surgeon and it also depends on the available instruments. The reports from the United Arab Emirates [44] and Nigeria [45] stated that open reduction and rigid internal fixation of the facial fractures have not become popular in most developing countries, mainly because of the cost issue [46]. In a study performed in Iran between 1987 and 2001, Ansari reported a marked predilection for ‘‘simple techniques’’ and most patients (70.8%) were treated by applying closed procedures [12]. Since 2004 in Iran, all costs of the management of trauma patients were covered by the government and the trend changed toward the use of internal rigid fixation.Our study showed that 68.8% of patients were treated by miniplates' osteosynthesis; only 21.9% of them were managed by closed techniques, confirming the effect of cost on the treatment planning.

## Conclusion

Isolated mandibular fracture due to road traffic accident was the most common type of maxillofacial injuries in the city of Shiraz. These findings should also alert the authorities, particularly the government to the need for the provision of good roads, enforcement of existing traffic laws and general improvement of the socioeconomic condition of the community.
